# Supplementary material for: Protein prediction models support widespread post-transcriptional regulation of protein abundance by interacting partners
Source: PLoS Comput Biol. 2022 Nov 10;18(11):e1010702. doi: 10.1371/journal.pcbi.1010702 (PMC9681107; doi:10.1371/journal.pcbi.1010702)
Supplement: S1 Fig — A. Box plots of test set normalized root mean square error (NRMSE) between the transcript-predicted and actual protein level for each protein are shown across five feature sets (column: single/self transcript, CORUM interactors, STRING high-confidence associated proteins; STRING low-confidence associated proteins, and all transcripts) and three algorithms (multiple linear regression, elastic net, and random forest). In each plot, x axis denotes the number of CPTAC data set used to train the models box: interquartile range; whiskers: +/– 1.5 IQR. B. As above, but for test set goodness-of-fit (R2). (PDF) [file pcbi.1010702.s001.pdf]

Supplementary Figures

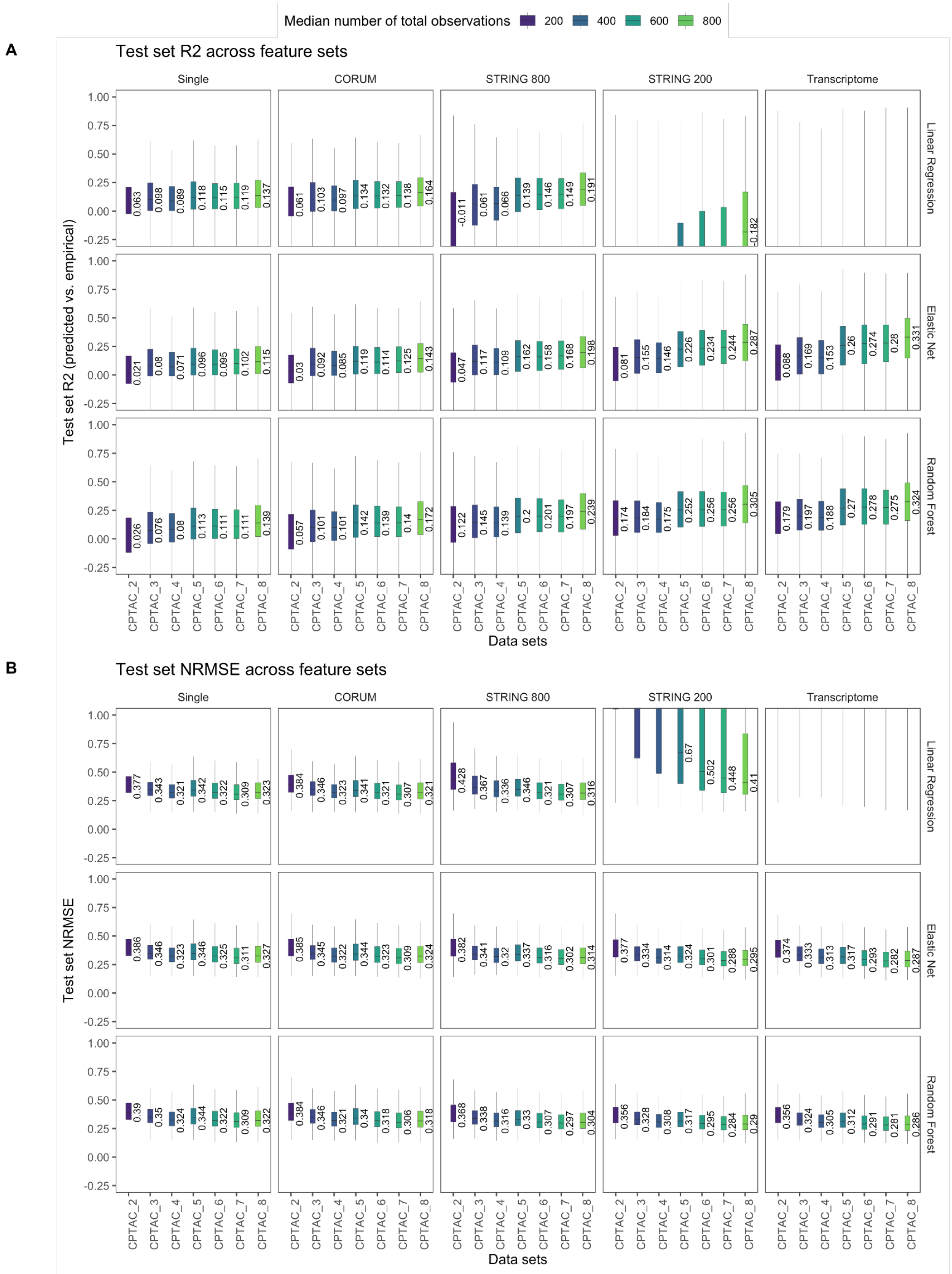

Supplementary Figure S1: Model performance measured by additional metrics. **A.** Box plots of test set normalized root mean square error (NRMSE) between the transcript-predicted and actual protein level for each protein are shown across five feature sets (column: single/self transcript, CORUM interactors, STRING high-confidence associated proteins; STRING low-confidence associated proteins, and all transcripts) and three algorithms (multiple linear regression, elastic net, and random forest). In each plot, x axis denotes the number of CPTAC data set used to train the models box: interquartile range; whiskers: +/– 1.5 IQR. **B.** As above, but for test set goodness-of-fit (R^2).
