# Supplementary material for: Protein prediction models support widespread post-transcriptional regulation of protein abundance by interacting partners
Source: PLoS Comput Biol. 2022 Nov 10;18(11):e1010702. doi: 10.1371/journal.pcbi.1010702 (PMC9681107; doi:10.1371/journal.pcbi.1010702)
Supplement: S2 Fig — A. Model performance in single cancer data sets. Box plots of test set correlation coefficients (r) between the transcript-predicted and actual protein level for each protein are shown across five feature sets (column: single/self transcript, CORUM interactors, STRING high-confidence associated proteins; STRING low-confidence associated proteins, and all transcripts) and three algorithms (multiple linear regression, elastic net, and random forest). In each plot, x axis denotes the CPTAC cancer type study used to train the models; box: interquartile range; whiskers: +/– 1.5 IQR. B. Model performance when the 8 data sets were combined in the order of decreasing single data set performance. (PDF) [file pcbi.1010702.s002.pdf]

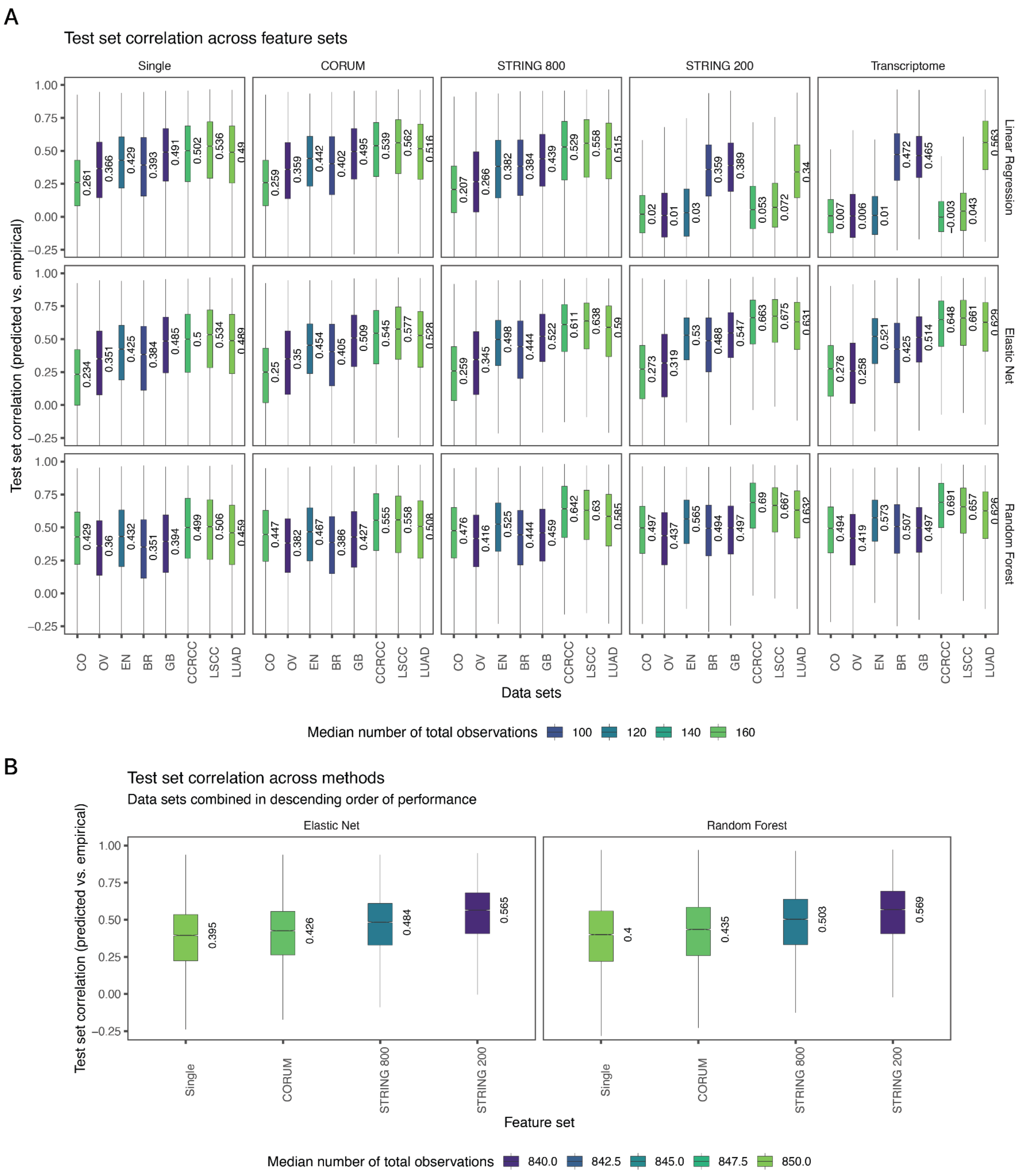

Supplementary Figure S2: A. Model performance in single cancer data sets. Box plots of test set correlation coefficients (r) between the transcript-predicted and actual protein level for each protein are

shown across five feature sets (column: single/self transcript, CORUM interactors, STRING high-confidence associated proteins; STRING low-confidence associated proteins, and all transcripts) and three algorithms (multiple linear regression, elastic net, and random forest). In each plot, x axis denotes the CPTAC cancer type study used to train the models; box: interquartile range; whiskers: +/– 1.5 IQR. **B.** Model performance when the 8 data sets were combined in the order of decreasing single data set performance.
