# Supplementary material for: Protein prediction models support widespread post-transcriptional regulation of protein abundance by interacting partners
Source: PLoS Comput Biol. 2022 Nov 10;18(11):e1010702. doi: 10.1371/journal.pcbi.1010702 (PMC9681107; doi:10.1371/journal.pcbi.1010702)
Supplement: S3 Fig — A–B. Scatterplot showing a significant linear relationship between the number of low-confidence interactors a protein has as annotated in STRING vs. the increase in test set correlation between predicted vs. actual protein levels in the Elastic Net models over the self-transcript feature set (correlation test p: 8.2e–131 for all proteins; 6.5e–130 for proteins with 10 or more interactors). C–D. As above, but for the CORUM feature set. (PDF) [file pcbi.1010702.s003.pdf]

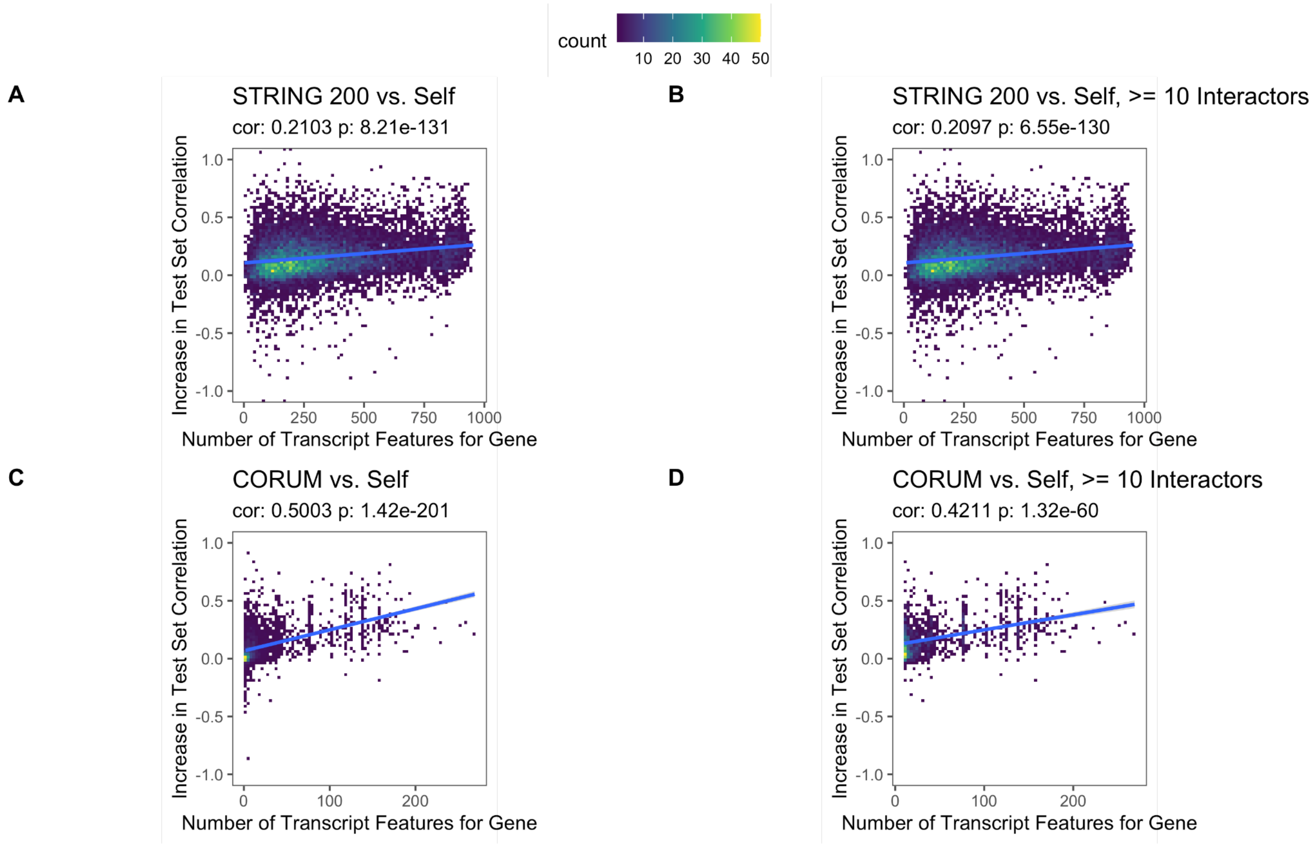

Supplementary Figure S3. Improvements to protein prediction from the incorporation of additional transcript features. A–B. Scatterplot showing a significant linear relationship between the number of low-confidence interactors a protein has as annotated in STRING vs. the increase in test set correlation between predicted vs. actual protein levels in the Elastic Net models over the self-transcript feature set (correlation test p: 8.2e–131 for all proteins; 6.5e–130 for proteins with 10 or more interactors). **C–D.** As above, but for the CORUM feature set.
