# Supplementary material for: Protein prediction models support widespread post-transcriptional regulation of protein abundance by interacting partners
Source: PLoS Comput Biol. 2022 Nov 10;18(11):e1010702. doi: 10.1371/journal.pcbi.1010702 (PMC9681107; doi:10.1371/journal.pcbi.1010702)
Supplement: S4 Fig — Two additional highlighted proteins with substantial predictability from transcriptome data upon the inclusion of additional features are shown: A. MICU2, B. PPP3R1. For each protein, the transcript-trained prediction of protein level is plotted on the x axis and the actual protein level is plotted on the y axis. Blue: train set, brown: test set. Columns denote the transcript feature set used to train the model. The number of features used to train the model in each feature set is shown inside each plot. r: Correlation coefficient. (PDF) [file pcbi.1010702.s004.pdf]

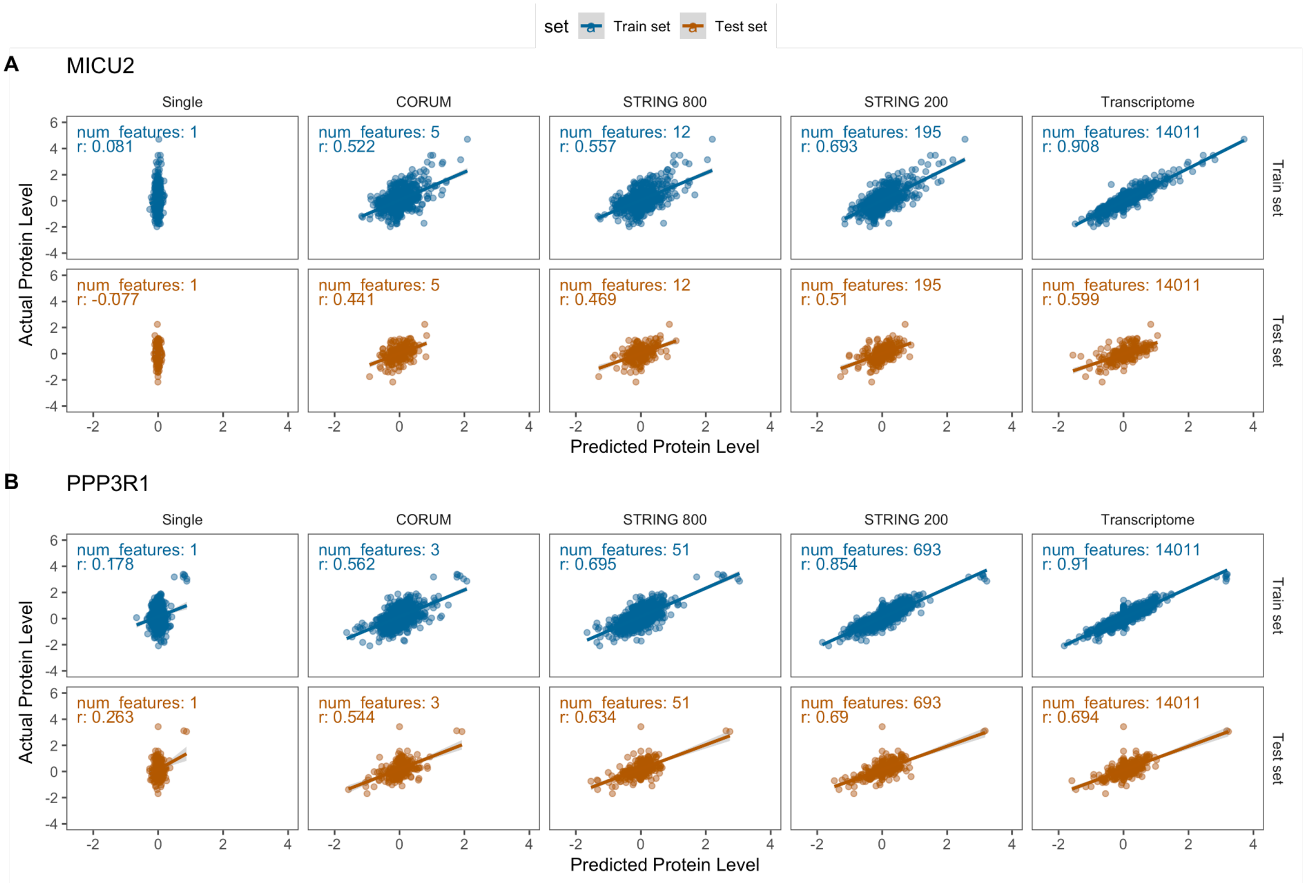

Supplementary Figure S4: Additional proteins with improved predicted levels after inclusion of additional transcript features. Two additional highlighted proteins with substantial predictability from transcriptome data upon the inclusion of additional features are shown: **A.** MICU2, **B.** PPP3R1. For each protein, the transcript-trained prediction of protein level is plotted on the x axis and the actual protein level is plotted on the y axis. Blue: train set, brown: test set. Columns denote the transcript feature set used to train the model. The number of features used to train the model in each feature set is shown inside each plot. r: Correlation coefficient.
