# Supplementary material for: Protein prediction models support widespread post-transcriptional regulation of protein abundance by interacting partners
Source: PLoS Comput Biol. 2022 Nov 10;18(11):e1010702. doi: 10.1371/journal.pcbi.1010702 (PMC9681107; doi:10.1371/journal.pcbi.1010702)
Supplement: S5 Fig — Two examples of proteins whose abundance is better explained by another transcript are shown. A. MICU2 protein level is predicted by MCU transcript but not its own transcript. B. PPP3R1 protein level is explained by PPP3CA transcript level but not its own transcript. Substantial correlations across transcripts and proteins (≥ 0.4) are bolded. (PDF) [file pcbi.1010702.s005.pdf]

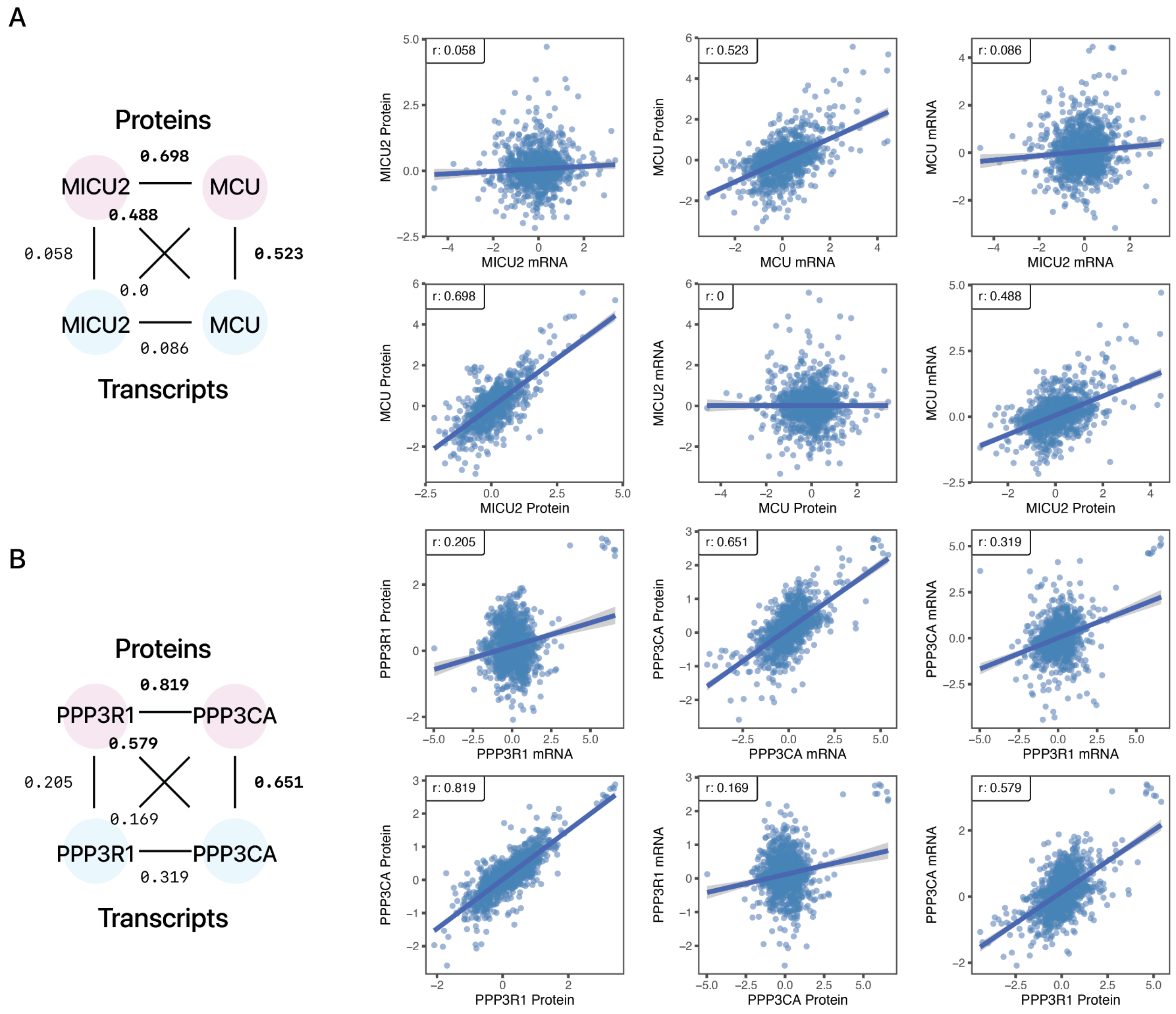

Supplementary Figure S5: Cross-omics co-expression of MICU2 and PPP3R1 with functionally associated proteins. Two examples of proteins whose abundance is better explained by another transcript are shown. **A.** MICU2 protein level is predicted by MCU transcript but not its own transcript. **B.** PPP3R1 protein level is explained by PPP3CA transcript level but not its own transcript. Substantial correlations across transcripts and proteins (≥ 0.4) are bolded.
