# Supplementary material for: Protein prediction models support widespread post-transcriptional regulation of protein abundance by interacting partners
Source: PLoS Comput Biol. 2022 Nov 10;18(11):e1010702. doi: 10.1371/journal.pcbi.1010702 (PMC9681107; doi:10.1371/journal.pcbi.1010702)
Supplement: S6 Fig — SHAP interpretation of feature importance in A. random forest and B. gradient boosting model output from the CPTAC_8 CORUM feature set. The SHAP values of top transcript features and their impact on model output are shown for each of six proteins highlighted in the text (from top to bottom): PCCB, CMC1, PSMG2, SMCR8, MICU2, PPP3R1. (PDF) [file pcbi.1010702.s006.pdf]

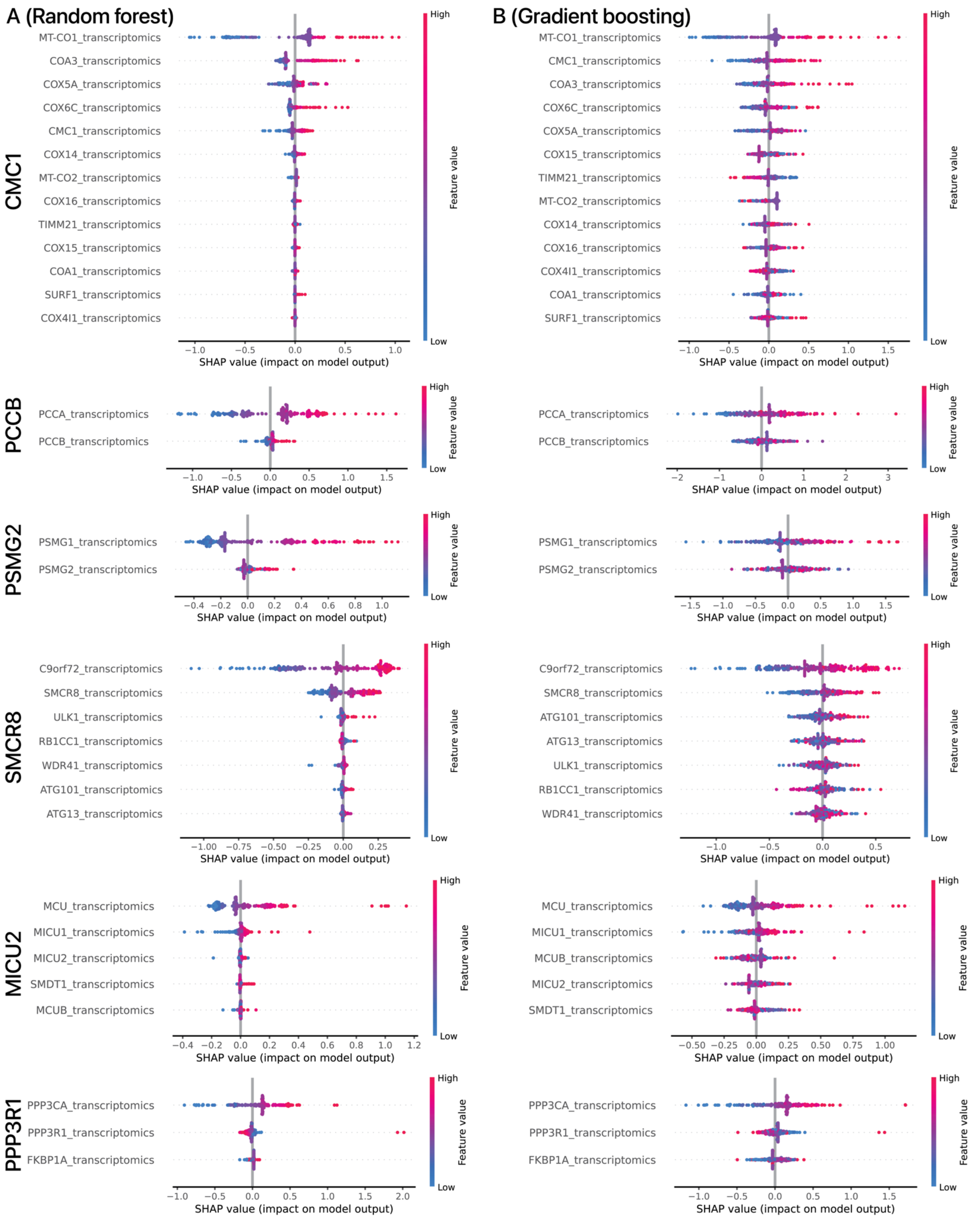

A (Random forest)
B (Gradient boosting)
CMC1
MT-CO1_transcriptomics
COA3_transcriptomics
COX5A_transcriptomics
COX6C_transcriptomics
CMC1_transcriptomics
COX14_transcriptomics
MT-CO2_transcriptomics
COX16_transcriptomics
TIMM21_transcriptomics
COX15_transcriptomics
COA1_transcriptomics
SURF1_transcriptomics
COX4I1_transcriptomics
PCCB
PCCA_transcriptomics
PCCB_transcriptomics
PSMG2
PSMG1_transcriptomics
PSMG2_transcriptomics
SMCR8
C9orf72_transcriptomics
SMCR8_transcriptomics
ULK1_transcriptomics
RB1CC1_transcriptomics
WDR41_transcriptomics
ATG101_transcriptomics
ATG13_transcriptomics
MICU2
MCU_transcriptomics
MICU1_transcriptomics
MICU2_transcriptomics
SMDT1_transcriptomics
MCUB_transcriptomics
PPP3R1
PPP3CA_transcriptomics
PPP3R1_transcriptomics
FKBP1A_transcriptomics
SHAP value (impact on model output)
Feature value
High
Low

Supplementary Figure S6: SHAP interpretation of feature importance in **A.** random forest and **B.** gradient boosting model output from the CPTAC_8 CORUM feature set. The SHAP values of top transcript features and their impact on model output are shown for each of six proteins highlighted in the text (from top to bottom): PCCB, CMC1, PSMG2, SMCR8, MICU2, PPP3R1.
