# Supplementary material for: Protein prediction models support widespread post-transcriptional regulation of protein abundance by interacting partners
Source: PLoS Comput Biol. 2022 Nov 10;18(11):e1010702. doi: 10.1371/journal.pcbi.1010702 (PMC9681107; doi:10.1371/journal.pcbi.1010702)
Supplement: S7 Fig — Consistent with the analysis of the combined CPTAC_8 data set, five of the six proteins are not best predicted by their cognate transcripts and all but one (MT-CO1) of the top trans locus transcripts are preserved, which was not among the examined candidate features. Only test set data points are shown. (PDF) [file pcbi.1010702.s007.pdf]

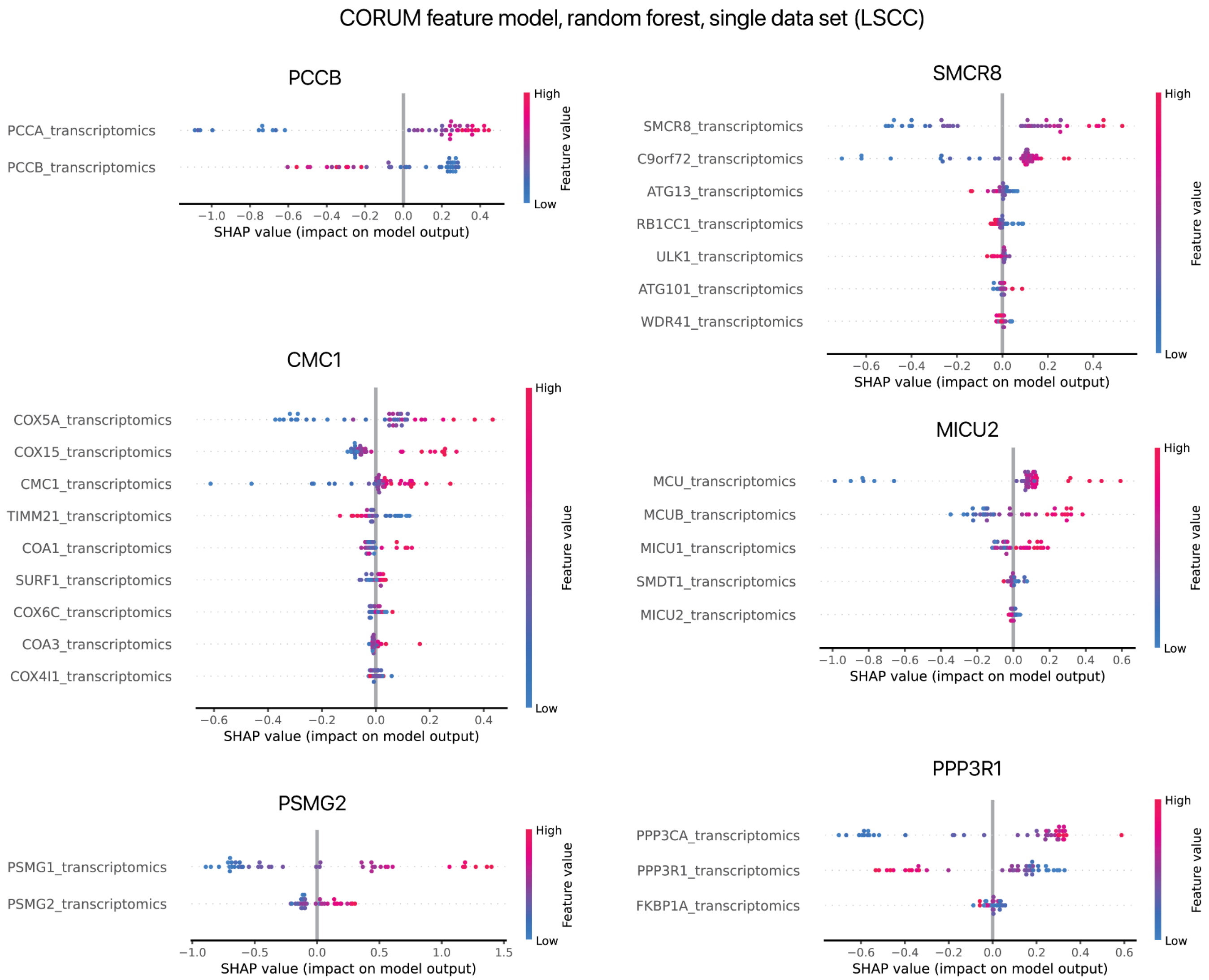

Supplementary Figure S7: SHAP interpretation of feature importance in the CORUM feature set, random forest model, of a single data set (LSCC), showing largely conserved observations among the highlighted proteins PCCB, CMC1, PSMG2, SMCR8, MICU2, PPP3R1. Consistent with the analysis of the combined CPTAC_8 data set, five of the six proteins are not best predicted by their cognate transcripts and all but one (MT-CO1) of the top trans locus transcripts are preserved, which was not among the examined candidate features. Only test set data points are shown.
