# Supplementary material for: Protein prediction models support widespread post-transcriptional regulation of protein abundance by interacting partners
Source: PLoS Comput Biol. 2022 Nov 10;18(11):e1010702. doi: 10.1371/journal.pcbi.1010702 (PMC9681107; doi:10.1371/journal.pcbi.1010702)
Supplement: S8 Fig — Names of select complexes are labeled. The majority of complexes are small with a median of 3 proteins per complex. (PDF) [file pcbi.1010702.s008.pdf]

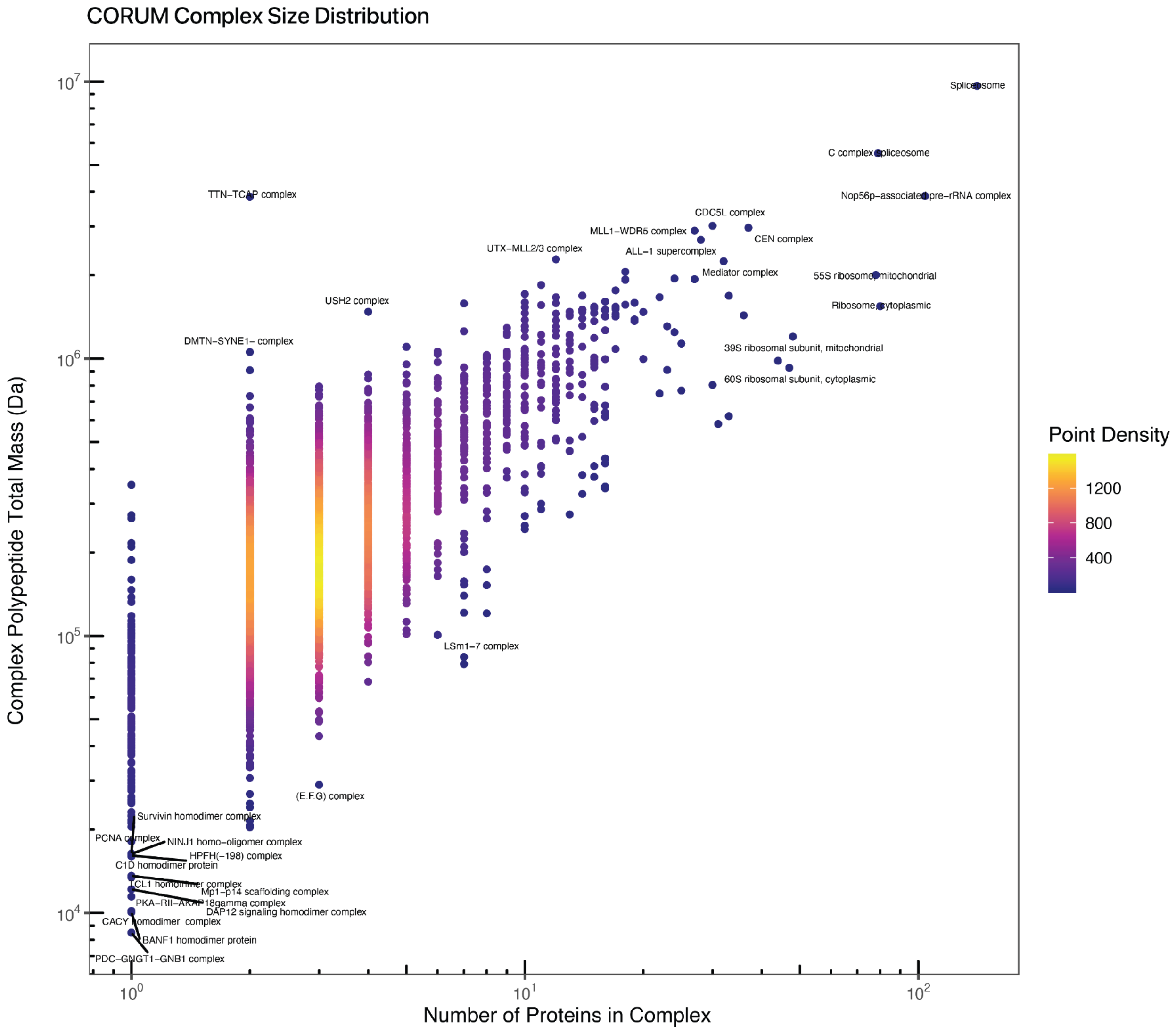

Supplementary Figure S8: Density plot showing the distribution of complex sizes (x:-axis: number of member sin complex; y-axis: log10 total polypeptide molecular weight in complex) in the annotated feature set derived from CORUM v.3.0. Names of select complexes are labeled. The majority of complexes are small with a median of 3 proteins per complex.
