# Supplementary material for: Protein prediction models support widespread post-transcriptional regulation of protein abundance by interacting partners
Source: PLoS Comput Biol. 2022 Nov 10;18(11):e1010702. doi: 10.1371/journal.pcbi.1010702 (PMC9681107; doi:10.1371/journal.pcbi.1010702)
Supplement: S9 Fig — The hierarchical network diagram shows Chromogranin A and B (CHGA/CHGB) (magenta) and their first-degree inward flow neighbors (green) in the largest subgraph constructed from the STRING data set. The protein level of CHGA and CHGB is associated with the transcript levels of multiple secreted peptide coding genes, consistent with these proteins serve to regulate gene expression in secretion pathways. (PDF) [file pcbi.1010702.s009.pdf]

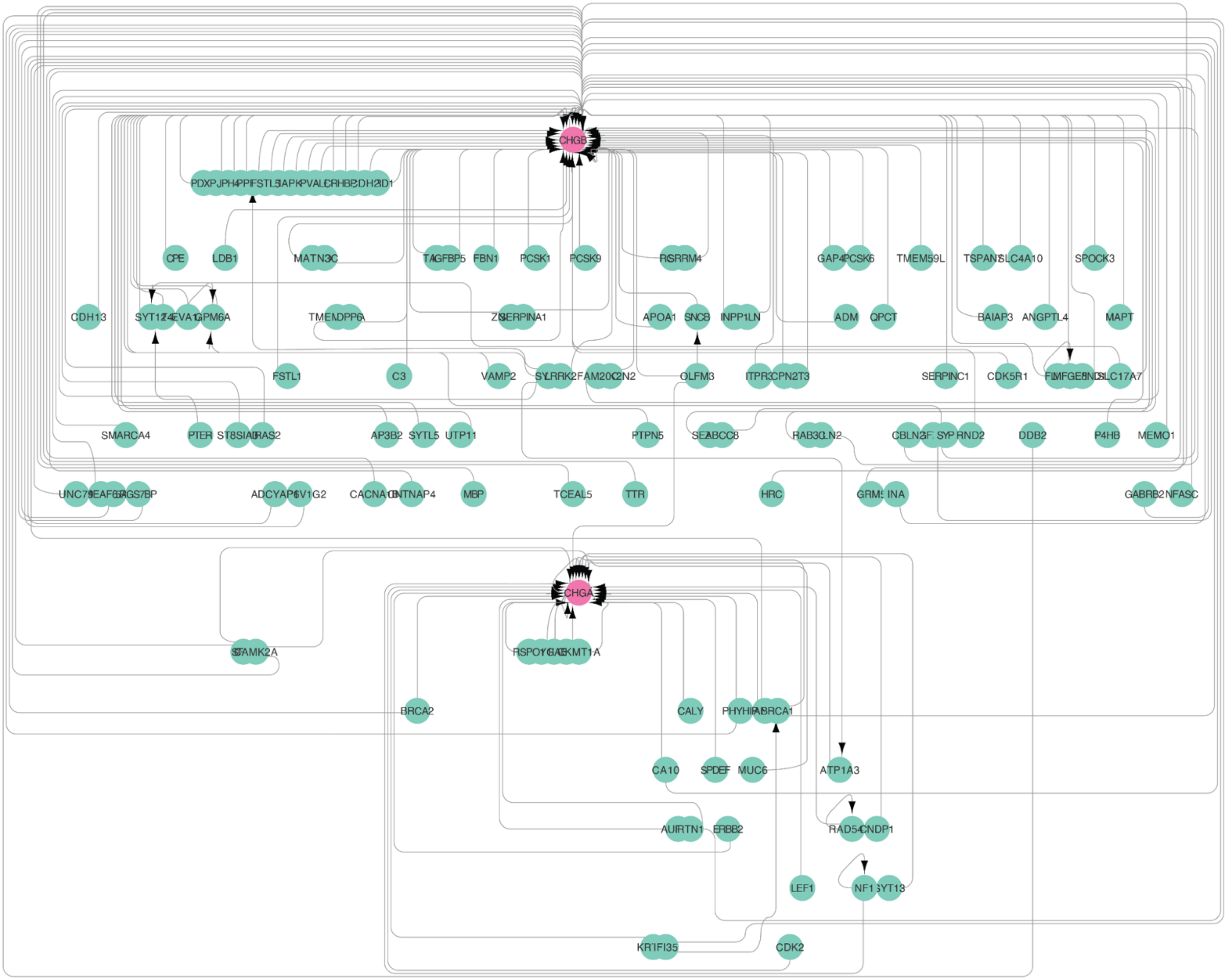

Supplementary Figure S9: The most connected subgraph in the STRING feature set contains 6,319 nodes and 11,161 edges, containing proteins belonging to multiple distinct cellular compartments and multi-protein complexes. The hierarchical network diagram shows Chromogranin A and B (CHGA/CHGB) (magenta) and their first-degree inward flow neighbors (green) in the largest subgraph constructed from the STRING data set. The protein level of CHGA and CHGB is associated with the transcript levels of multiple secreted peptide coding genes, consistent with these proteins serve to regulate gene expression in secretion pathways.
